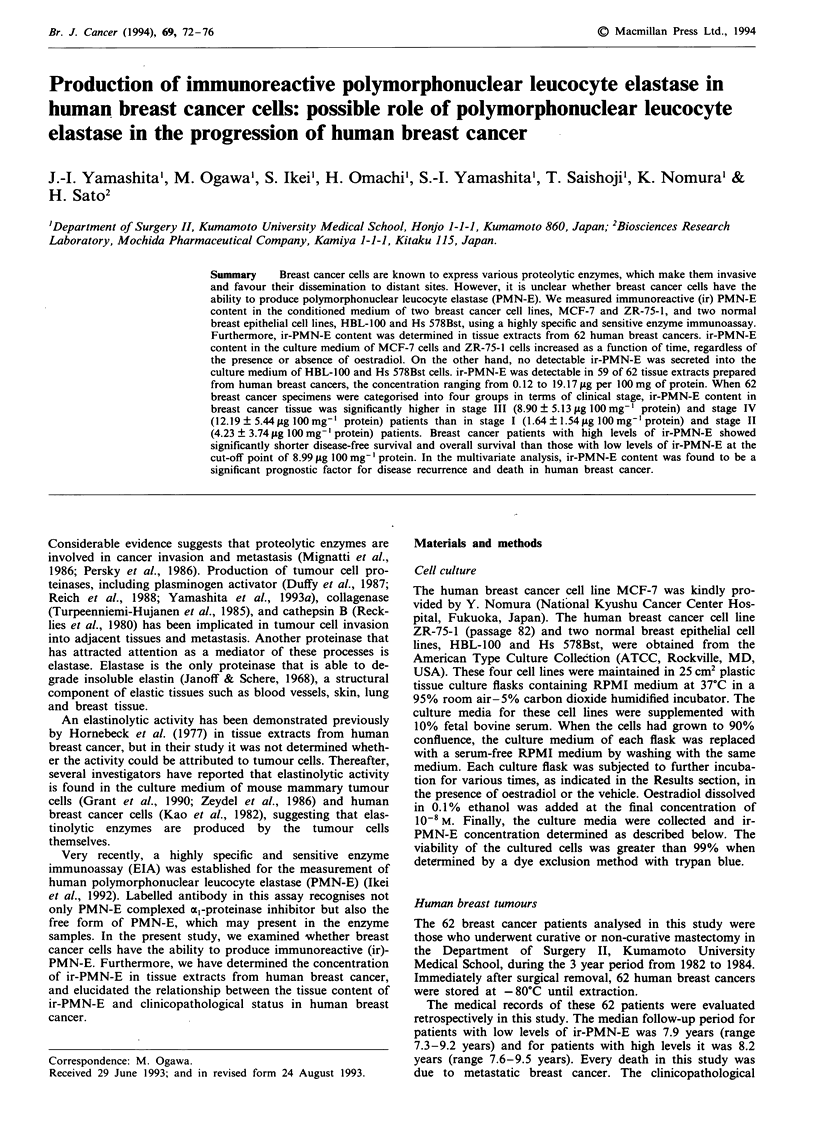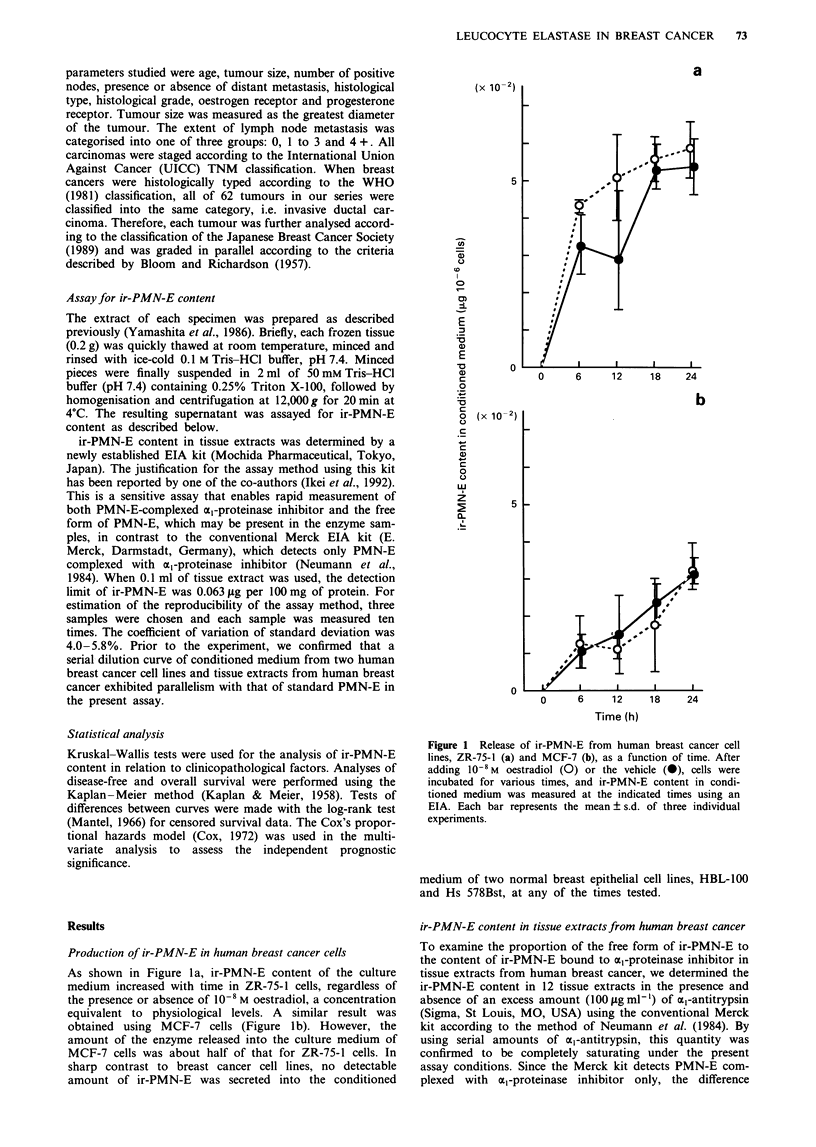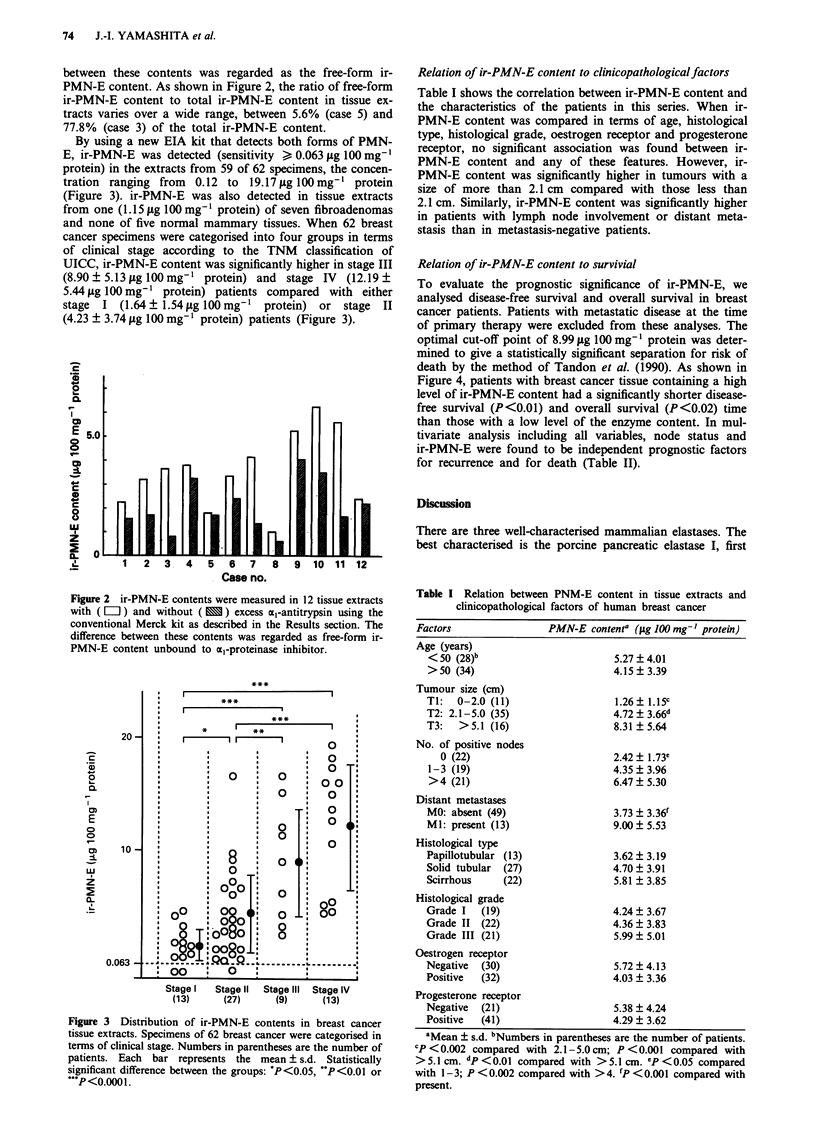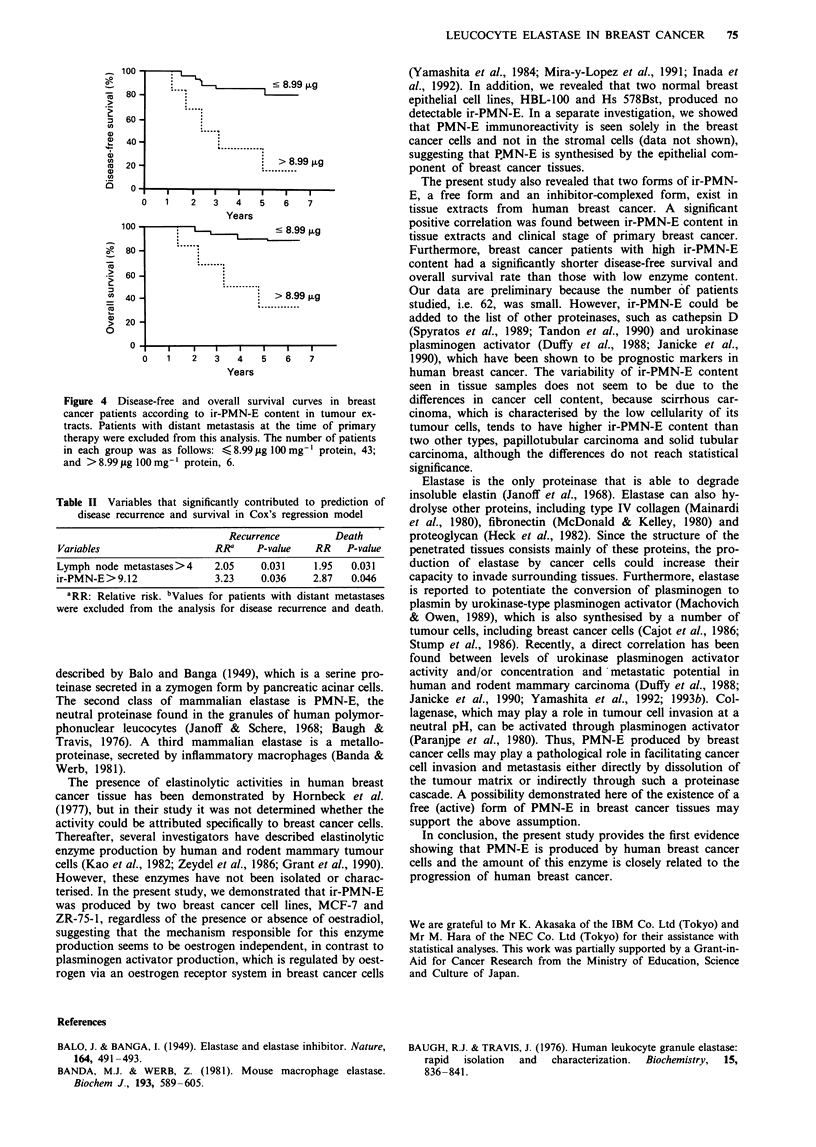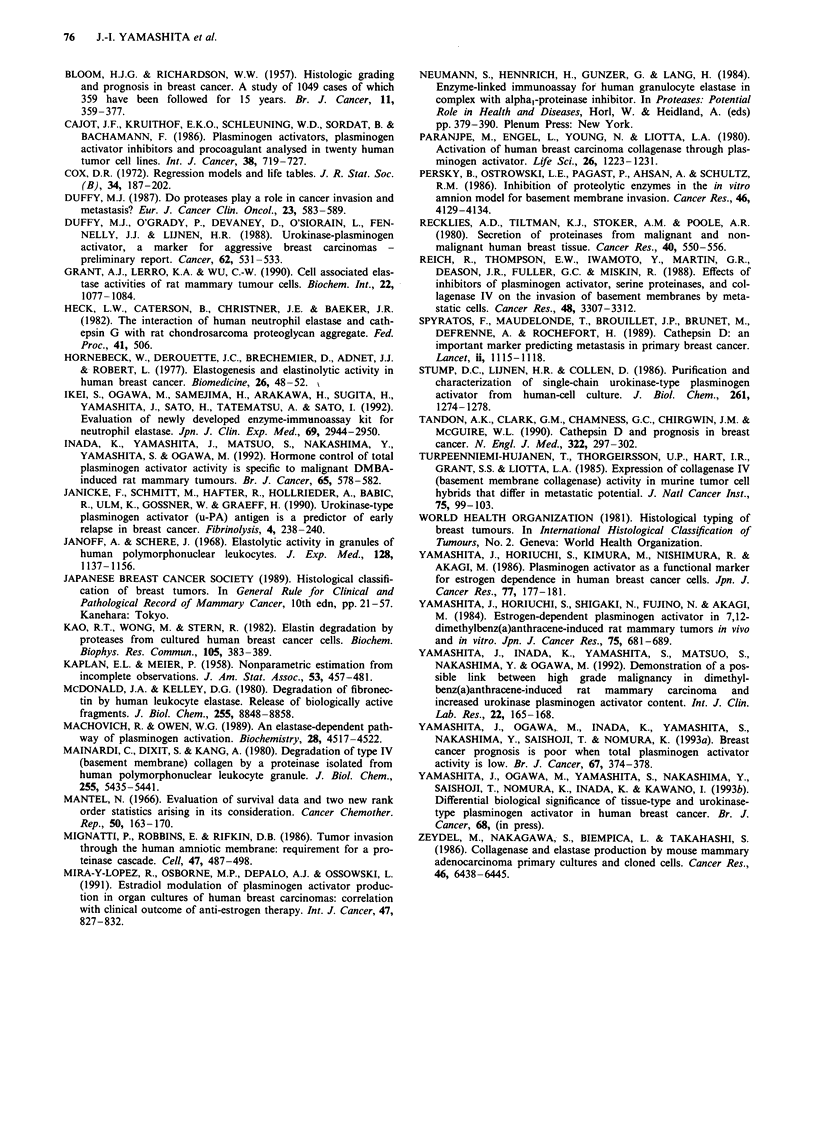# Production of immunoreactive polymorphonuclear leucocyte elastase in human breast cancer cells: possible role of polymorphonuclear leucocyte elastase in the progression of human breast cancer.

**DOI:** 10.1038/bjc.1994.11

**Published:** 1994-01

**Authors:** J. I. Yamashita, M. Ogawa, S. Ikei, H. Omachi, S. I. Yamashita, T. Saishoji, K. Nomura, H. Sato

**Affiliations:** Department of Surgery II, Kumamoto University Medical School, Japan.

## Abstract

Breast cancer cells are known to express various proteolytic enzymes, which make them invasive and favour their dissemination to distant sites. However, it is unclear whether breast cancer cells have the ability to produce polymorphonuclear leucocyte elastase (PMN-E). We measured immunoreactive (ir) PMN-E content in the conditioned medium of two breast cancer cell lines, MCF-7 and ZR-75-1, and two normal breast epithelial cell lines, HBL-100 and Hs 578Bst, using a highly specific and sensitive enzyme immunoassay. Furthermore, ir-PMN-E content was determined in tissue extracts from 62 human breast cancers. ir-PMN-E content in the culture medium of MCF-7 cells and ZR-75-1 cells increased as a function of time, regardless of the presence or absence of oestradiol. On the other hand, no detectable ir-PMN-E was secreted into the culture medium of HBL-100 and Hs 578Bst cells. ir-PMN-E was detectable in 59 of 62 tissue extracts prepared from human breast cancers, the concentration ranging from 0.12 to 19.17 micrograms per 100 mg of protein. When 62 breast cancer specimens were categorised into four groups in terms of clinical stage, ir-PMN-E content in breast cancer tissue was significantly higher in stage III (8.90 +/- 5.13 micrograms 100 mg-1 protein) and stage IV (12.19 +/- 5.44 micrograms 100 mg-1 protein) patients than in stage I (1.64 +/- 1.54 micrograms 100 mg-1 protein) and stage II (4.23 +/- 3.74 micrograms 100 mg-1 protein) patients. Breast cancer patients with high levels of ir-PMN-E showed significantly shorter disease-free survival and overall survival than those with low levels of ir-PMN-E at the cut-off point of 8.99 micrograms 100 mg-1 protein. In the multivariate analysis, ir-PMN-E content was found to be a significant prognostic factor for disease recurrence and death in human breast cancer.


Br. J. Cancer (1994), 69, 72-76 © Macmillan Press Ltd., 1994

### Production of immunoreactive polymorphonuclear leucocyte elastase in human breast cancer cells: possible role of polymorphonuclear leucocyte elastase in the progression of human breast cancer

J.-I. Yamashita[1], M. Ogawa[1], S. Ikei[1], H. Omachi[1], S.-I. Yamashita[1], T. Saishoji[1], K. Nomura[1] & H. Sato[2]

[1]*Department of Surgery II, Kumamoto University Medical School, Honjo 1-1-1, Kumamoto 860, Japan;* [2]*Biosciences Research Laboratory, Mochida Pharmaceutical Company, Kamiya 1-1-1, Kitaku 115, Japan.*

**Summary** Breast cancer cells are known to express various proteolytic enzymes, which make them invasive and favour their dissemination to distant sites. However, it is unclear whether breast cancer cells have the ability to produce polymorphonuclear leucocyte elastase (PMN-E). We measured immunoreactive (ir) PMN-E content in the conditioned medium of two breast cancer cell lines, MCF-7 and ZR-75-1, and two normal breast epithelial cell lines, HBL-100 and Hs 578Bst, using a highly specific and sensitive enzyme immunoassay. Furthermore, ir-PMN-E content was determined in tissue extracts from 62 human breast cancers. ir-PMN-E content in the culture medium of MCF-7 cells and ZR-75-1 cells increased as a function of time, regardless of the presence or absence of oestradiol. On the other hand, no detectable ir-PMN-E was secreted into the culture medium of HBL-100 and Hs 578Bst cells. ir-PMN-E was detectable in 59 of 62 tissue extracts prepared from human breast cancers, the concentration ranging from 0.12 to 19.17 μg per 100 mg of protein. When 62 breast cancer specimens were categorised into four groups in terms of clinical stage, ir-PMN-E content in breast cancer tissue was significantly higher in stage III ($8.90 \pm 5.13$ μg 100 $mg^{-1}$ protein) and stage IV ($12.19 \pm 5.44$ μg 100 $mg^{-1}$ protein) patients than in stage I ($1.64 \pm 1.54$ μg 100 $mg^{-1}$ protein) and stage II ($4.23 \pm 3.74$ μg 100 $mg^{-1}$ protein) patients. Breast cancer patients with high levels of ir-PMN-E showed significantly shorter disease-free survival and overall survival than those with low levels of ir-PMN-E at the cut-off point of 8.99 μg 100 $mg^{-1}$ protein. In the multivariate analysis, ir-PMN-E content was found to be a significant prognostic factor for disease recurrence and death in human breast cancer.

Considerable evidence suggests that proteolytic enzymes are involved in cancer invasion and metastasis (Mignatti *et al.*, 1986; Persky *et al.*, 1986). Production of tumour cell proteinases, including plasminogen activator (Duffy *et al.*, 1987; Reich *et al.*, 1988; Yamashita *et al.*, 1993*a*), collagenase (Turpeenniemi-Hujanen *et al.*, 1985), and cathepsin B (Recklies *et al.*, 1980) has been implicated in tumour cell invasion into adjacent tissues and metastasis. Another proteinase that has attracted attention as a mediator of these processes is elastase. Elastase is the only proteinase that is able to degrade insoluble elastin (Janoff & Schere, 1968), a structural component of elastic tissues such as blood vessels, skin, lung and breast tissue.

An elastinolytic activity has been demonstrated previously by Hornebeck *et al.* (1977) in tissue extracts from human breast cancer, but in their study it was not determined whether the activity could be attributed to tumour cells. Thereafter, several investigators have reported that elastinolytic activity is found in the culture medium of mouse mammary tumour cells (Grant *et al.*, 1990; Zeydel *et al.*, 1986) and human breast cancer cells (Kao *et al.*, 1982), suggesting that elastinolytic enzymes are produced by the tumour cells themselves.

Very recently, a highly specific and sensitive enzyme immunoassay (EIA) was established for the measurement of human polymorphonuclear leucocyte elastase (PMN-E) (Ikei *et al.*, 1992). Labelled antibody in this assay recognises not only PMN-E complexed $\alpha_1$-proteinase inhibitor but also the free form of PMN-E, which may present in the enzyme samples. In the present study, we examined whether breast cancer cells have the ability to produce immunoreactive (ir)-PMN-E. Furthermore, we have determined the concentration of ir-PMN-E in tissue extracts from human breast cancer, and elucidated the relationship between the tissue content of ir-PMN-E and clinicopathological status in human breast cancer.

Correspondence: M. Ogawa.
Received 29 June 1993; and in revised form 24 August 1993.

### Materials and methods

### *Cell culture*

The human breast cancer cell line MCF-7 was kindly provided by Y. Nomura (National Kyushu Cancer Center Hospital, Fukuoka, Japan). The human breast cancer cell line ZR-75-1 (passage 82) and two normal breast epithelial cell lines, HBL-100 and Hs 578Bst, were obtained from the American Type Culture Collection (ATCC, Rockville, MD, USA). These four cell lines were maintained in 25 $cm^2$ plastic tissue culture flasks containing RPMI medium at 37°C in a 95% room air-5% carbon dioxide humidified incubator. The culture media for these cell lines were supplemented with 10% fetal bovine serum. When the cells had grown to 90% confluence, the culture medium of each flask was replaced with a serum-free RPMI medium by washing with the same medium. Each culture flask was subjected to further incubation for various times, as indicated in the Results section, in the presence of oestradiol or the vehicle. Oestradiol dissolved in 0.1% ethanol was added at the final concentration of $10^{-8}$ M. Finally, the culture media were collected and ir-PMN-E concentration determined as described below. The viability of the cultured cells was greater than 99% when determined by a dye exclusion method with trypan blue.

### *Human breast tumours*

The 62 breast cancer patients analysed in this study were those who underwent curative or non-curative mastectomy in the Department of Surgery II, Kumamoto University Medical School, during the 3 year period from 1982 to 1984. Immediately after surgical removal, 62 human breast cancers were stored at $-80$°C until extraction.

The medical records of these 62 patients were evaluated retrospectively in this study. The median follow-up period for patients with low levels of ir-PMN-E was 7.9 years (range 7.3-9.2 years) and for patients with high levels it was 8.2 years (range 7.6-9.5 years). Every death in this study was due to metastatic breast cancer. The clinicopathological parameters studied were age, tumour size, number of positive nodes, presence or absence of distant metastasis, histological type, histological grade, oestrogen receptor and progesterone receptor. Tumour size was measured as the greatest diameter of the tumour. The extent of lymph node metastasis was categorised into one of three groups: 0, 1 to 3 and 4 +. All carcinomas were staged according to the International Union Against Cancer (UICC) TNM classification. When breast cancers were histologically typed according to the WHO (1981) classification, all of 62 tumours in our series were classified into the same category, i.e. invasive ductal carcinoma. Therefore, each tumour was further analysed according to the classification of the Japanese Breast Cancer Society (1989) and was graded in parallel according to the criteria described by Bloom and Richardson (1957).

### *Assay for ir-PMN-E content*

The extract of each specimen was prepared as described previously (Yamashita *et al.*, 1986). Briefly, each frozen tissue (0.2 g) was quickly thawed at room temperature, minced and rinsed with ice-cold 0.1 M Tris–HCl buffer, pH 7.4. Minced pieces were finally suspended in 2 ml of 50 mM Tris–HCl buffer (pH 7.4) containing 0.25% Triton X-100, followed by homogenisation and centrifugation at 12,000 *g* for 20 min at 4°C. The resulting supernatant was assayed for ir-PMN-E content as described below.

ir-PMN-E content in tissue extracts was determined by a newly established EIA kit (Mochida Pharmaceutical, Tokyo, Japan). The justification for the assay method using this kit has been reported by one of the co-authors (Ikei *et al.*, 1992). This is a sensitive assay that enables rapid measurement of both PMN-E-complexed $\alpha_1$-proteinase inhibitor and the free form of PMN-E, which may be present in the enzyme samples, in contrast to the conventional Merck EIA kit (E. Merck, Darmstadt, Germany), which detects only PMN-E complexed with $\alpha_1$-proteinase inhibitor (Neumann *et al.*, 1984). When 0.1 ml of tissue extract was used, the detection limit of ir-PMN-E was 0.063 µg per 100 mg of protein. For estimation of the reproducibility of the assay method, three samples were chosen and each sample was measured ten times. The coefficient of variation of standard deviation was 4.0–5.8%. Prior to the experiment, we confirmed that a serial dilution curve of conditioned medium from two human breast cancer cell lines and tissue extracts from human breast cancer exhibited parallelism with that of standard PMN-E in the present assay.

### *Statistical analysis*

Kruskal–Wallis tests were used for the analysis of ir-PMN-E content in relation to clinicopathological factors. Analyses of disease-free and overall survival were performed using the Kaplan–Meier method (Kaplan & Meier, 1958). Tests of differences between curves were made with the log-rank test (Mantel, 1966) for censored survival data. The Cox's proportional hazards model (Cox, 1972) was used in the multivariate analysis to assess the independent prognostic significance.

### Results

### *Production of ir-PMN-E in human breast cancer cells*

As shown in Figure 1a, ir-PMN-E content of the culture medium increased with time in ZR-75-1 cells, regardless of the presence or absence of $10^{-8}$ M oestradiol, a concentration equivalent to physiological levels. A similar result was obtained using MCF-7 cells (Figure 1b). However, the amount of the enzyme released into the culture medium of MCF-7 cells was about half of that for ZR-75-1 cells. In sharp contrast to breast cancer cell lines, no detectable amount of ir-PMN-E was secreted into the conditioned

**Figure 1** Release of ir-PMN-E from human breast cancer cell lines, ZR-75-1 (**a**) and MCF-7 (**b**), as a function of time. After adding $10^{-8}$ M oestradiol (○) or the vehicle (●), cells were incubated for various times, and ir-PMN-E content in conditioned medium was measured at the indicated times using an EIA. Each bar represents the mean ± s.d. of three individual experiments.

medium of two normal breast epithelial cell lines, HBL-100 and Hs 578Bst, at any of the times tested.

### *ir-PMN-E content in tissue extracts from human breast cancer*

To examine the proportion of the free form of ir-PMN-E to the content of ir-PMN-E bound to $\alpha_1$-proteinase inhibitor in tissue extracts from human breast cancer, we determined the ir-PMN-E content in 12 tissue extracts in the presence and absence of an excess amount (100 µg ml$^{-1}$) of $\alpha_1$-antitrypsin (Sigma, St Louis, MO, USA) using the conventional Merck kit according to the method of Neumann *et al.* (1984). By using serial amounts of $\alpha_1$-antitrypsin, this quantity was confirmed to be completely saturating under the present assay conditions. Since the Merck kit detects PMN-E complexed with $\alpha_1$-proteinase inhibitor only, the difference between these contents was regarded as the free-form ir-PMN-E content. As shown in Figure 2, the ratio of free-form ir-PMN-E content to total ir-PMN-E content in tissue extracts varies over a wide range, between 5.6% (case 5) and 77.8% (case 3) of the total ir-PMN-E content.

By using a new EIA kit that detects both forms of PMN-E, ir-PMN-E was detected (sensitivity $\geqslant 0.063\,\mu g\,100\,mg^{-1}$ protein) in the extracts from 59 of 62 specimens, the concentration ranging from 0.12 to $19.17\,\mu g\,100\,mg^{-1}$ protein (Figure 3). ir-PMN-E was also detected in tissue extracts from one ($1.15\,\mu g\,100\,mg^{-1}$ protein) of seven fibroadenomas and none of five normal mammary tissues. When 62 breast cancer specimens were categorised into four groups in terms of clinical stage according to the TNM classification of UICC, ir-PMN-E content was significantly higher in stage III ($8.90 \pm 5.13\,\mu g\,100\,mg^{-1}$ protein) and stage IV ($12.19 \pm 5.44\,\mu g\,100\,mg^{-1}$ protein) patients compared with either stage I ($1.64 \pm 1.54\,\mu g\,100\,mg^{-1}$ protein) or stage II ($4.23 \pm 3.74\,\mu g\,100\,mg^{-1}$ protein) patients (Figure 3).

**Figure 2** ir-PMN-E contents were measured in 12 tissue extracts with ( ☐ ) and without ( ▨ ) excess $\alpha_1$-antitrypsin using the conventional Merck kit as described in the Results section. The difference between these contents was regarded as free-form ir-PMN-E content unbound to $\alpha_1$-proteinase inhibitor.

**Figure 3** Distribution of ir-PMN-E contents in breast cancer tissue extracts. Specimens of 62 breast cancer were categorised in terms of clinical stage. Numbers in parentheses are the number of patients. Each bar represents the mean ± s.d. Statistically significant difference between the groups: *$P<0.05$, **$P<0.01$ or ***$P<0.0001$.

*Relation of ir-PMN-E content to clinicopathological factors*

Table I shows the correlation between ir-PMN-E content and the characteristics of the patients in this series. When ir-PMN-E content was compared in terms of age, histological type, histological grade, oestrogen receptor and progesterone receptor, no significant association was found between ir-PMN-E content and any of these features. However, ir-PMN-E content was significantly higher in tumours with a size of more than 2.1 cm compared with those less than 2.1 cm. Similarly, ir-PMN-E content was significantly higher in patients with lymph node involvement or distant metastasis than in metastasis-negative patients.

*Relation of ir-PMN-E content to survivial*

To evaluate the prognostic significance of ir-PMN-E, we analysed disease-free survival and overall survival in breast cancer patients. Patients with metastatic disease at the time of primary therapy were excluded from these analyses. The optimal cut-off point of $8.99\,\mu g\,100\,mg^{-1}$ protein was determined to give a statistically significant separation for risk of death by the method of Tandon *et al.* (1990). As shown in Figure 4, patients with breast cancer tissue containing a high level of ir-PMN-E content had a significantly shorter disease-free survival ($P<0.01$) and overall survival ($P<0.02$) time than those with a low level of the enzyme content. In multivariate analysis including all variables, node status and ir-PMN-E were found to be independent prognostic factors for recurrence and for death (Table II).

### Discussion

There are three well-characterised mammalian elastases. The best characterised is the porcine pancreatic elastase I, first

**Table I** Relation between PNM-E content in tissue extracts and clinicopathological factors of human breast cancer

| *Factors* | *PMN-E content*[a] *(µg 100 mg⁻¹ protein)* |
|---|---|
| Age (years) | |
| <50 (28)[b] | 5.27 ± 4.01 |
| >50 (34) | 4.15 ± 3.39 |
| Tumour size (cm) | |
| T1: 0–2.0 (11) | 1.26 ± 1.15[c] |
| T2: 2.1–5.0 (35) | 4.72 ± 3.66[d] |
| T3: >5.1 (16) | 8.31 ± 5.64 |
| No. of positive nodes | |
| 0 (22) | 2.42 ± 1.73[e] |
| 1–3 (19) | 4.35 ± 3.96 |
| >4 (21) | 6.47 ± 5.30 |
| Distant metastases | |
| M0: absent (49) | 3.73 ± 3.36[f] |
| M1: present (13) | 9.00 ± 5.53 |
| Histological type | |
| Papillotubular (13) | 3.62 ± 3.19 |
| Solid tubular (27) | 4.70 ± 3.91 |
| Scirrhous (22) | 5.81 ± 3.85 |
| Histological grade | |
| Grade I (19) | 4.24 ± 3.67 |
| Grade II (22) | 4.36 ± 3.83 |
| Grade III (21) | 5.99 ± 5.01 |
| Oestrogen receptor | |
| Negative (30) | 5.72 ± 4.13 |
| Positive (32) | 4.03 ± 3.36 |
| Progesterone receptor | |
| Negative (21) | 5.38 ± 4.24 |
| Positive (41) | 4.29 ± 3.62 |

[a]Mean ± s.d. [b]Numbers in parentheses are the number of patients. [c]$P<0.002$ compared with 2.1–5.0 cm; $P<0.001$ compared with >5.1 cm. [d]$P<0.01$ compared with >5.1 cm. [e]$P<0.05$ compared with 1–3; $P<0.002$ compared with >4. [f]$P<0.001$ compared with present.

**Figure 4** Disease-free and overall survival curves in breast cancer patients according to ir-PMN-E content in tumour extracts. Patients with distant metastasis at the time of primary therapy were excluded from this analysis. The number of patients in each group was as follows: $\leqslant 8.99\,\mu g\ 100\,mg^{-1}$ protein, 43; and $>8.99\,\mu g\ 100\,mg^{-1}$ protein, 6.

**Table II** Variables that significantly contributed to prediction of disease recurrence and survival in Cox's regression model

| | *Recurrence* | | *Death* | |
|---|---|---|---|---|
| *Variables* | *RR*[a] | *P-value* | *RR* | *P-value* |
| Lymph node metastases > 4 | 2.05 | 0.031 | 1.95 | 0.031 |
| ir-PMN-E > 9.12 | 3.23 | 0.036 | 2.87 | 0.046 |

[a]RR: Relative risk. [b]Values for patients with distant metastases were excluded from the analysis for disease recurrence and death.

described by Balo and Banga (1949), which is a serine proteinase secreted in a zymogen form by pancreatic acinar cells. The second class of mammalian elastase is PMN-E, the neutral proteinase found in the granules of human polymorphonuclear leucocytes (Janoff & Schere, 1968; Baugh & Travis, 1976). A third mammalian elastase is a metalloproteinase, secreted by inflammatory macrophages (Banda & Werb, 1981).

The presence of elastinolytic activities in human breast cancer tissue has been demonstrated by Hornbeck *et al.* (1977), but in their study it was not determined whether the activity could be attributed specifically to breast cancer cells. Thereafter, several investigators have described elastinolytic enzyme production by human and rodent mammary tumour cells (Kao *et al.*, 1982; Zeydel *et al.*, 1986; Grant *et al.*, 1990). However, these enzymes have not been isolated or characterised. In the present study, we demonstrated that ir-PMN-E was produced by two breast cancer cell lines, MCF-7 and ZR-75-1, regardless of the presence or absence of oestradiol, suggesting that the mechanism responsible for this enzyme production seems to be oestrogen independent, in contrast to plasminogen activator production, which is regulated by oestrogen via an oestrogen receptor system in breast cancer cells (Yamashita *et al.*, 1984; Mira-y-Lopez *et al.*, 1991; Inada *et al.*, 1992). In addition, we revealed that two normal breast epithelial cell lines, HBL-100 and Hs 578Bst, produced no detectable ir-PMN-E. In a separate investigation, we showed that PMN-E immunoreactivity is seen solely in the breast cancer cells and not in the stromal cells (data not shown), suggesting that PMN-E is synthesised by the epithelial component of breast cancer tissues.

The present study also revealed that two forms of ir-PMN-E, a free form and an inhibitor-complexed form, exist in tissue extracts from human breast cancer. A significant positive correlation was found between ir-PMN-E content in tissue extracts and clinical stage of primary breast cancer. Furthermore, breast cancer patients with high ir-PMN-E content had a significantly shorter disease-free survival and overall survival rate than those with low enzyme content. Our data are preliminary because the number of patients studied, i.e. 62, was small. However, ir-PMN-E could be added to the list of other proteinases, such as cathepsin D (Spyratos *et al.*, 1989; Tandon *et al.*, 1990) and urokinase plasminogen activator (Duffy *et al.*, 1988; Janicke *et al.*, 1990), which have been shown to be prognostic markers in human breast cancer. The variability of ir-PMN-E content seen in tissue samples does not seem to be due to the differences in cancer cell content, because scirrhous carcinoma, which is characterised by the low cellularity of its tumour cells, tends to have higher ir-PMN-E content than two other types, papillotubular carcinoma and solid tubular carcinoma, although the differences do not reach statistical significance.

Elastase is the only proteinase that is able to degrade insoluble elastin (Janoff *et al.*, 1968). Elastase can also hydrolyse other proteins, including type IV collagen (Mainardi *et al.*, 1980), fibronectin (McDonald & Kelley, 1980) and proteoglycan (Heck *et al.*, 1982). Since the structure of the penetrated tissues consists mainly of these proteins, the production of elastase by cancer cells could increase their capacity to invade surrounding tissues. Furthermore, elastase is reported to potentiate the conversion of plasminogen to plasmin by urokinase-type plasminogen activator (Machovich & Owen, 1989), which is also synthesised by a number of tumour cells, including breast cancer cells (Cajot *et al.*, 1986; Stump *et al.*, 1986). Recently, a direct correlation has been found between levels of urokinase plasminogen activator activity and/or concentration and metastatic potential in human and rodent mammary carcinoma (Duffy *et al.*, 1988; Janicke *et al.*, 1990; Yamashita *et al.*, 1992; 1993*b*). Collagenase, which may play a role in tumour cell invasion at a neutral pH, can be activated through plasminogen activator (Paranjpe *et al.*, 1980). Thus, PMN-E produced by breast cancer cells may play a pathological role in facilitating cancer cell invasion and metastasis either directly by dissolution of the tumour matrix or indirectly through such a proteinase cascade. A possibility demonstrated here of the existence of a free (active) form of PMN-E in breast cancer tissues may support the above assumption.

In conclusion, the present study provides the first evidence showing that PMN-E is produced by human breast cancer cells and the amount of this enzyme is closely related to the progression of human breast cancer.

We are grateful to Mr K. Akasaka of the IBM Co. Ltd (Tokyo) and Mr M. Hara of the NEC Co. Ltd (Tokyo) for their assistance with statistical analyses. This work was partially supported by a Grant-in-Aid for Cancer Research from the Ministry of Education, Science and Culture of Japan.

### References

BALO, J. & BANGA, I. (1949). Elastase and elastase inhibitor. *Nature*, **164**, 491–493.

BANDA, M.J. & WERB, Z. (1981). Mouse macrophage elastase. *Biochem J.*, **193**, 589–605.

BAUGH, R.J. & TRAVIS, J. (1976). Human leukocyte granule elastase: rapid isolation and characterization. *Biochemistry*, **15**, 836–841.

BLOOM, H.J.G. & RICHARDSON, W.W. (1957). Histologic grading and prognosis in breast cancer. A study of 1049 cases of which 359 have been followed for 15 years. *Br. J. Cancer*, **11**, 359–377.

CAJOT, J.F., KRUITHOF, E.K.O., SCHLEUNING, W.D., SORDAT, B. & BACHAMANN, F. (1986). Plasminogen activators, plasminogen activator inhibitors and procoagulant analysed in twenty human tumor cell lines. *Int. J. Cancer*, **38**, 719–727.

COX, D.R. (1972). Regression models and life tables. *J. R. Stat. Soc. (B)*, **34**, 187–202.

DUFFY, M.J. (1987). Do proteases play a role in cancer invasion and metastasis? *Eur. J. Cancer Clin. Oncol.*, **23**, 583–589.

DUFFY, M.J., O'GRADY, P., DEVANEY, D., O'SIORAIN, L., FENNELLY, J.J. & LIJNEN, H.R. (1988). Urokinase-plasminogen activator, a marker for aggressive breast carcinomas – preliminary report. *Cancer*, **62**, 531–533.

GRANT, A.J., LERRO, K.A. & WU, C.-W. (1990). Cell associated elastase activities of rat mammary tumour cells. *Biochem. Int.*, **22**, 1077–1084.

HECK, L.W., CATERSON, B., CHRISTNER, J.E. & BAEKER, J.R. (1982). The interaction of human neutrophil elastase and cathepsin G with rat chondrosarcoma proteoglycan aggregate. *Fed. Proc.*, **41**, 506.

HORNEBECK, W., DEROUETTE, J.C., BRECHEMIER, D., ADNET, J.J. & ROBERT, L. (1977). Elastogenesis and elastinolytic activity in human breast cancer. *Biomedicine*, **26**, 48–52.

IKEI, S., OGAWA, M., SAMEJIMA, H., ARAKAWA, H., SUGITA, H., YAMASHITA, J., SATO, H., TATEMATSU, A. & SATO, I. (1992). Evaluation of newly developed enzyme-immunoassay kit for neutrophil elastase. *Jpn. J. Clin. Exp. Med.*, **69**, 2944–2950.

INADA, K., YAMASHITA, J., MATSUO, S., NAKASHIMA, Y., YAMASHITA, S. & OGAWA, M. (1992). Hormone control of total plasminogen activator activity is specific to malignant DMBA-induced rat mammary tumours. *Br. J. Cancer*, **65**, 578–582.

JANICKE, F., SCHMITT, M., HAFTER, R., HOLLRIEDER, A., BABIC, R., ULM, K., GOSSNER, W. & GRAEFF, H. (1990). Urokinase-type plasminogen activator (u-PA) antigen is a predictor of early relapse in breast cancer. *Fibrinolysis*, **4**, 238–240.

JANOFF, A. & SCHERE, J. (1968). Elastolytic activity in granules of human polymorphonuclear leukocytes. *J. Exp. Med.*, **128**, 1137–1156.

JAPANESE BREAST CANCER SOCIETY (1989). Histological classification of breast tumors. In *General Rule for Clinical and Pathological Record of Mammary Cancer*, 10th edn, pp. 21–57. Kanehara: Tokyo.

KAO, R.T., WONG, M. & STERN, R. (1982). Elastin degradation by proteases from cultured human breast cancer cells. *Biochem. Biophys. Res. Commun.*, **105**, 383–389.

KAPLAN, E.L. & MEIER, P. (1958). Nonparametric estimation from incomplete observations. *J. Am. Stat. Assoc.*, **53**, 457–481.

MCDONALD, J.A. & KELLEY, D.G. (1980). Degradation of fibronectin by human leukocyte elastase. Release of biologically active fragments. *J. Biol. Chem.*, **255**, 8848–8858.

MACHOVICH, R. & OWEN, W.G. (1989). An elastase-dependent pathway of plasminogen activation. *Biochemistry*, **28**, 4517–4522.

MAINARDI, C., DIXIT, S. & KANG, A. (1980). Degradation of type IV (basement membrane) collagen by a proteinase isolated from human polymorphonuclear leukocyte granule. *J. Biol. Chem.*, **255**, 5435–5441.

MANTEL, N. (1966). Evaluation of survival data and two new rank order statistics arising in its consideration. *Cancer Chemother. Rep.*, **50**, 163–170.

MIGNATTI, P., ROBBINS, E. & RIFKIN, D.B. (1986). Tumor invasion through the human amniotic membrane: requirement for a proteinase cascade. *Cell*, **47**, 487–498.

MIRA-Y-LOPEZ, R., OSBORNE, M.P., DEPALO, A.J. & OSSOWSKI, L. (1991). Estradiol modulation of plasminogen activator production in organ cultures of human breast carcinomas: correlation with clinical outcome of anti-estrogen therapy. *Int. J. Cancer*, **47**, 827–832.

NEUMANN, S., HENNRICH, H., GUNZER, G. & LANG, H. (1984). Enzyme-linked immunoassay for human granulocyte elastase in complex with $alpha_1$-proteinase inhibitor. In *Proteases: Potential Role in Health and Diseases*, Horl, W. & Heidland, A. (eds) pp. 379–390. Plenum Press: New York.

PARANJPE, M., ENGEL, L., YOUNG, N. & LIOTTA, L.A. (1980). Activation of human breast carcinoma collagenase through plasminogen activator. *Life Sci.*, **26**, 1223–1231.

PERSKY, B., OSTROWSKI, L.E., PAGAST, P., AHSAN, A. & SCHULTZ, R.M. (1986). Inhibition of proteolytic enzymes in the *in vitro* amnion model for basement membrane invasion. *Cancer Res.*, **46**, 4129–4134.

RECKLIES, A.D., TILTMAN, K.J., STOKER, A.M. & POOLE, A.R. (1980). Secretion of proteinases from malignant and nonmalignant human breast tissue. *Cancer Res.*, **40**, 550–556.

REICH, R., THOMPSON, E.W., IWAMOTO, Y., MARTIN, G.R., DEASON, J.R., FULLER, G.C. & MISKIN, R. (1988). Effects of inhibitors of plasminogen activator, serine proteinases, and collagenase IV on the invasion of basement membranes by metastatic cells. *Cancer Res.*, **48**, 3307–3312.

SPYRATOS, F., MAUDELONDE, T., BROUILLET, J.P., BRUNET, M., DEFRENNE, A. & ROCHEFORT, H. (1989). Cathepsin D: an important marker predicting metastasis in primary breast cancer. *Lancet*, **ii**, 1115–1118.

STUMP, D.C., LIJNEN, H.R. & COLLEN, D. (1986). Purification and characterization of single-chain urokinase-type plasminogen activator from human-cell culture. *J. Biol. Chem.*, **261**, 1274–1278.

TANDON, A.K., CLARK, G.M., CHAMNESS, G.C., CHIRGWIN, J.M. & MCGUIRE, W.L. (1990). Cathepsin D and prognosis in breast cancer. *N. Engl. J. Med.*, **322**, 297–302.

TURPEENNIEMI-HUJANEN, T., THORGEIRSSON, U.P., HART, I.R., GRANT, S.S. & LIOTTA, L.A. (1985). Expression of collagenase IV (basement membrane collagenase) activity in murine tumor cell hybrids that differ in metastatic potential. *J. Natl Cancer Inst.*, **75**, 99–103.

WORLD HEALTH ORGANIZATION (1981). Histological typing of breast tumours. In *International Histological Classification of Tumours*, No. 2. Geneva: World Health Organization.

YAMASHITA, J., HORIUCHI, S., KIMURA, M., NISHIMURA, R. & AKAGI, M. (1986). Plasminogen activator as a functional marker for estrogen dependence in human breast cancer cells. *Jpn. J. Cancer Res.*, **77**, 177–181.

YAMASHITA, J., HORIUCHI, S., SHIGAKI, N., FUJINO, N. & AKAGI, M. (1984). Estrogen-dependent plasminogen activator in 7,12-dimethylbenz(a)anthracene-induced rat mammary tumors *in vivo* and *in vitro*. *Jpn. J. Cancer Res.*, **75**, 681–689.

YAMASHITA, J., INADA, K., YAMASHITA, S., MATSUO, S., NAKASHIMA, Y. & OGAWA, M. (1992). Demonstration of a possible link between high grade malignancy in dimethylbenz(a)anthracene-induced rat mammary carcinoma and increased urokinase plasminogen activator content. *Int. J. Clin. Lab. Res.*, **22**, 165–168.

YAMASHITA, J., OGAWA, M., INADA, K., YAMASHITA, S., NAKASHIMA, Y., SAISHOJI, T. & NOMURA, K. (1993*a*). Breast cancer prognosis is poor when total plasminogen activator activity is low. *Br. J. Cancer*, **67**, 374–378.

YAMASHITA, J., OGAWA, M., YAMASHITA, S., NAKASHIMA, Y., SAISHOJI, T., NOMURA, K., INADA, K. & KAWANO, I. (1993*b*). Differential biological significance of tissue-type and urokinase-type plasminogen activator in human breast cancer. *Br. J. Cancer*, **68**, (in press).

ZEYDEL, M., NAKAGAWA, S., BIEMPICA, L. & TAKAHASHI, S. (1986). Collagenase and elastase production by mouse mammary adenocarcinoma primary cultures and cloned cells. *Cancer Res.*, **46**, 6438–6445.